# Interventions to improve linear growth during exclusive breastfeeding life-stage for children aged 0-6 months living in low- and middle-income countries: a systematic review with network and pairwise meta-analyses

**DOI:** 10.12688/gatesopenres.13082.2

**Published:** 2020-09-24

**Authors:** Jay J. H. Park, Ellie Siden, Ofir Harari, Louis Dron, Reham Mazoub, Virginia Jeziorska, Noor-E Zannat, Heather Gadalla, Kristian Thorlund, Edward J. Mills

**Affiliations:** 1 MTEK Sciences, Vancouver, BC, V5Z1J5, Canada; 2 Department of Medicine, University of British Columbia, Vancouver, BC, V6T 1Z4, Canada; 3 Department of Health Research Methodology, Evidence, and Impact, McMaster University, Hamilton, ON, L8S4K1, Canada

**Keywords:** Exclusive breastfeeding, linear growth, stunting, low- and middle-income countries, network meta-analysis

## Abstract

**Background: **Exclusive breastfeeding (EBF) during the first six months of life is critical for child’s linear growth. While there is strong evidence in favor of EBF, the evidence with regards to other interventions for linear growth is unclear. We evaluated intervention domains of micronutrients, food supplements, deworming, maternal education, water sanitation and hygiene (WASH), and kangaroo care, for their comparative effectiveness on linear growth.

**Methods:** For this review, we searched for randomized clinical trials (RCTs) of the interventions provided to infants aged 0-6 months and/or their breastfeeding mothers in low- and middle-income countries reporting on length-for-age z-score (LAZ), stunting, length, and head circumference. We searched for reports published until September 17 ^th^, 2019 and hand-searched bibliographies of existing reviews. For LAZ and stunting, we used network meta-analysis (NMA) to compare the effects of all interventions except for kangaroo care, where we used pairwise meta-analysis to compare its effects versus standard-of-care. For length and head circumference, we qualitatively summarized our findings.

**Results:** We found 29 RCTs (40 papers) involving 35,119 mother and infant pairs reporting on the effects of aforementioned interventions on linear growth outcomes. Our NMA on LAZ found that compared to standard-of-care, multiple micronutrients administered to infants (MMN-C) improved LAZ (mean difference: 0.20; 95% credible interval [CrI]: 0.03,0.35), whereas supplementing breastfeeding mothers with MMN did not (MMN-M, mean difference: -0.02, 95%CrI: -0.18,0.13). No interventions including MMN-C (relative risk: 0.74; 95%CrI: 0.36,1.44) reduced risk for stunting compared to standard-of-care. Kangaroo care, on the other hand, improved head circumference (mean difference: 0.20 cm/week; 95% confidence intervals [CI]: 0.09,0.31 cm/week) and length (mean difference: 0.23 cm/week; 95%CI: 0.10,0.35 cm/week) compared to standard-of-care.

**Conclusion:** Our study found important improvements for kangaroo care, but we did not find sufficient evidence for other interventions.

**Registration: **PROSPERO CRD42018110450; registered on 17 October 2018.

## Introduction

In past decades, important progress achieved in maternal, newborn, and child health (MNCH) have led to substantial reductions in maternal and child mortality rates ^1, 2^. However, many children still fail to reach their linear growth potential, particularly those living in low- and middle-income countries (LMICs) ^3^. Linear growth in early childhood is a marker of healthy development that is closely linked with neurodevelopment ^4^. The first six months of age (birth to 6 months) is a critical life stage for early child development, and exclusive breastfeeding during this life stage plays an important role in impacting the growth velocity of children. There is a strong evidence to support the benefits of exclusive breastfeeding during this life stage which includes protection against gastrointestinal infection, growth faltering, infant death syndrome etc. ^5– 7^. Exclusively breastfed children aged between zero to six months are known to have better growth rates and immune system. As such, mechanisms and resources to facilitate appropriate self-care in addition to psycho-social support for breastfeeding mothers is necessary to improve both health outcomes of mothers and babies. For instance, poor maternal nutrition could lead to lactation issues creating barriers for mothers to exclusively breastfeed ^3^. Inadequate care, poor hygiene, and control of diseases for infants and mothers may also inadvertently limit the growth of infants who are adequately breastfed ^3, 8, 9^.

The current evidence for other interventions, such as micronutrients, food supplements, deworming, maternal education, and kangaroo care (i.e. early skin-to-skin care) interventions is unclear for the exclusive breastfeeding life stage. Although there are numerous published reviews aimed to assess the effectiveness of these interventions that can be provided during exclusive breastfeeding period ( Table 1), their scope has been limited to summarize the comparative effectiveness of a single intervention or interventions within a single domain only. Given that determinants of linear growth for exclusive breastfeeding period is multi-faceted, there is a need to summarize the evidence base of interventions from multiple intervention domains, since multi-domain intervention solutions are likely needed to tackle this problem.

**Table 1.** Existing reviews on interventions for exclusive breastfeeding period.

Review ID	Title	Interventions	No of studies	Included study types
Kramer 2012 ^5^	Optimal duration of exclusive breastfeeding (Review).	Exclusive breastfeeding vs complementary food introduction at 4 months	23	Randomized trials
Lumbiganon 2016 ^13^	Antenatal breastfeeding education for increasing breastfeeding duration.	Breastfeeding education for increasing breastfeeding duration	24	Randomized trials
Haroon 2013 ^14^	Breastfeeding promotion interventions and breastfeeding practices: a systematic review.	Breastfeeding education or support	110	Randomized trials and quasi- experimental studies
Balogun 2016 ^15^	Interventions for promoting the initiation of breastfeeding.	Breastfeeding education, support groups	28	Randomized trials
Giugliani 2015 ^16^	Effect of breastfeeding promotion interventions on child growth: a systematic review and meta- analysis.	Breastfeeding promoting interventions	35	Randomized trials
Abe 2016 ^17^	Supplementation with multiple micronutrients for breastfeeding women for improving outcomes for the mother and baby.	Micronutrients mothers	2	Randomized trials
Ndikom 2014 ^18^	Extra fluids for breastfeeding mothers for increasing milk production.	Forced fluids	1	Randomized trials
Martin 2016 ^19^	Review of Infant Feeding: Key Features of Breast Milk and Infant Formula.	Infant nutrition	6	Randomized trials
Fleith 2005 ^20^	Dietary PUFA for Preterm and Term Infants: Review of Clinical Studies	Infant nutrition	28	Randomized trials
Conde-Agudelo 2016 ^21^	Kangaroo mother care to reduce morbidity and mortality in low birthweight infants.	Kangaroo care	21	Randomized trials
Moore 2016 ^22^	Early skin-to-skin contact for mothers and their healthy newborn infants.	Kangaroo care	46	Randomized trials
Delgado- Noguera 2015 ^23^	Supplementation with long chain polyunsaturated fatty acids (LCPUFA) to breastfeeding mothers for improving child growth and development.	Long chain polyunsaturated fatty acids supplements	8	Randomized trials
Thiele 2013 ^24^	Maternal vitamin D supplementation to meet the needs of the breastfed infant: a systematic review.	Vitamin D supplements	3	Randomized trials
Becker 2016 ^25^	Methods of milk expression for lactating women.	Methods of lactation	41	Randomized trials

This article uses a comprehensive literature review for multiple intervention domains of micronutrient, food supplements, deworming, maternal education, water sanitation and hygiene (WASH), and kangaroo care to summarize their effects on linear growth for LMIC-based infants in the exclusive breastfeeding period. For our quantitative summary, we have used network meta-analysis for all interventions except for kangaroo care to summarize their effects on LAZ and stunting outcomes; kangaroo care was assessed using pairwise meta-analysis. As the data was too sparse to facilitate meta-analysis, we qualitatively summarized the evidence base for outcomes length and head circumference.

## Methods

Our analysis and report was designed and reported according to the Preferred Reporting Items for Systematic Reviews and Meta-Analysis (PRISMA) extension to network meta-analysis ^10^. The protocol for this study was registered in PROSPERO ( CRD42018110450).

### Search strategy and selection criteria

Our search strategy was developed after first reviewing the papers published in the Lancet 2013 Maternal and Child Nutrition series ^3, 11^, inclusive of the umbrella review by Bhutta and colleagues ^7^, for an overview of the literature. Specifically, we hand-searched the bibliography of Bhutta *et al.* ^7^ for relevant systematic reviews, global health guidelines, and LMIC-based trials. We also performed additional searches in PubMed and the Cochrane Database of Systematic Reviews for more recent trials and other reviews published after 2013. The list of published reviews relevant to this study is provided in Table 1.

For our systematic literature search, we scanned the following databases from inception to August 28, 2019: the Cochrane Central Register of Controlled Trials, Embase, and MEDLINE ( *Extended data*, Supplementary Tables 1–3) ^12^. To increase the sensitivity of our search, we complemented our database searches with relevant trials identified from bibliographies of prior reviews. Table 2 describes the Population, Intervention, Comparator, Outcome, and Study Design (PICOS) criteria used to guide the study selection for our systematic literature review. We included randomized clinical trials on interventions of the following domains: Micronutrient supplements; Food supplements; Deworming for mothers; Maternal and breastfeeding education, and promotion; WASH; and kangaroo care (i.e. skin-to-skin care). The outcomes of interest included change in LAZ, proportions of participants with stunting (defined by LAZ below -2SD), change in length, and change in head circumference. We used LAZ reported from the individual trials that were calculated using the WHO Child Growth Standards ^26^. For all intervention domains, except for kangaroo care, we excluded studies that did not report the effects of their respective interventions for at least two months. For kangaroo care, there was no restriction for time of follow-up given short duration nature of this intervention. We excluded non-English language studies.

**Table 2.** Population, interventions, comparator, outcomes, and study design criteria.

Category	Inclusion criteria
Population	Infants of age 0 to 6 months, living in low- and middle-income countries
Intervention	• Micronutrient & calcium supplementation to mothers or infants • Food supplementation to mothers or infants • Kangaroo care* • Deworming • Maternal and breastfeeding education and promotion • Water, sanitation and hygiene (WASH) intervention
Comparators	• Placebo • Standard-of-care (if applicable) • No intervention • Any of the interventions listed above as monotherapy or in combination that can be used for indirect comparison
Outcomes	At least one of the following outcomes (reported after at least 2 months, *except for kangaroo care): • Length for age z-score (LAZ) • Proportion of stunted (LAZ < -2SD) • Length or height • Head circumference
Study Design	Randomized clinical trials
Other	Published in the English language

A team of four reviewers (JJHP, ES, LD, and RM) independently reviewed all abstracts and proceedings identified in the literature searches. The same team independently conducted relevant full-text reviews of relevant papers. If any discrepancies occurred between the studies selected by the same reviewers, a third investigator (KT) provided arbitration.

Using a standardized data sheet in Microsoft Excel, four investigators (JJHP, VJ, NEZ, and HG) independently extracted data for study characteristics, interventions used, subject characteristics at baseline, and outcomes from the final list of selected eligible studies. Any discrepancies observed during data extraction were resolved through discussion between the investigators until consensus was reached.

### Evidence synthesis and data analysis

When sufficient data was available for quantitative assessment, a network meta-analysis or pairwise meta-analysis approach was applied. For all domains of interventions except for kangaroo care, we performed a network meta-analysis for LAZ and stunting. There was a limited number of studies that reported on length and head circumference, so we qualitatively synthesized findings from these trials as an alternative to quantitative analysis. We did not consider kangaroo care as part of the network meta-analysis since these trials involved a shorter intervention duration and follow-up (median follow-up of 2 weeks). 

We performed a network meta-analysis within the Bayesian framework in R using the R2WinBUGS v14 package ^27, 28^. Bayesian models were performed according to the National Institute for Health and Care Excellence (NICE) in their Technical Support Document 2 (TSD2) ^29^. Estimates of comparative effectiveness were measured using mean differences in LAZ with the associated 95% credible intervals (95% CrI). In all models, we used an empirically informative heterogeneity prior distribution, as suggested by Rhodes *et al.* 2016 ^30^ for LAZ and Turner *et al.* 2015 ^31^ for stunting. This was done to stabilize the estimation of heterogeneity in the face of low number of trials per comparison in the network. Our model selection was informed by using the deviance information criterion and the deviance-leverage plots that could help identify outlier(s) in terms of model fit, in accordance with the NICE TSD2 recommendations ^29^.

For our primary network meta-analysis, we included both cluster and non-cluster randomized clinical trials (with the unit of randomization set at the individual level). To adjust for clustering effects of the cluster trials, we assumed a conservative intra-cluster correlation coefficient (ICC) of 0.05, and we inflated variances accordingly for continuous outcomes and adjusted the sample sizes and the number of cases for dichotomous outcomes, as recommended by Uhlmann *et al.* ^32^ We performed a sensitivity analysis by excluding cluster randomized clinical trials in our network meta-analysis. For our pairwise meta-analysis on kangaroo care, we performed a random-effects model using the Metafor R package (in R2WinBUGS v14) ^33^. For our network meta-analysis, the estimates of effectiveness were measured using mean differences or relative risk with accompanying 95% credible intervals (CrIs). The estimates of effectiveness were measured using mean differences with accompanying 95% confidence intervals (CIs) for our pairwise meta-analysis on kangaroo care. As no kangaroo care trials involved cluster randomization, our pairwise meta-analysis did not need to adjust for the clustering effect.

### Risk of bias within and across studies

Each full text article was evaluated for reporting quality according to the Cochrane Risk of Bias Tool ^34^. The risk of bias assessment within and across studies are provided in the *Extended data* (Supplementary Table 8) ^12^. 

## Results

**Figure 1.**
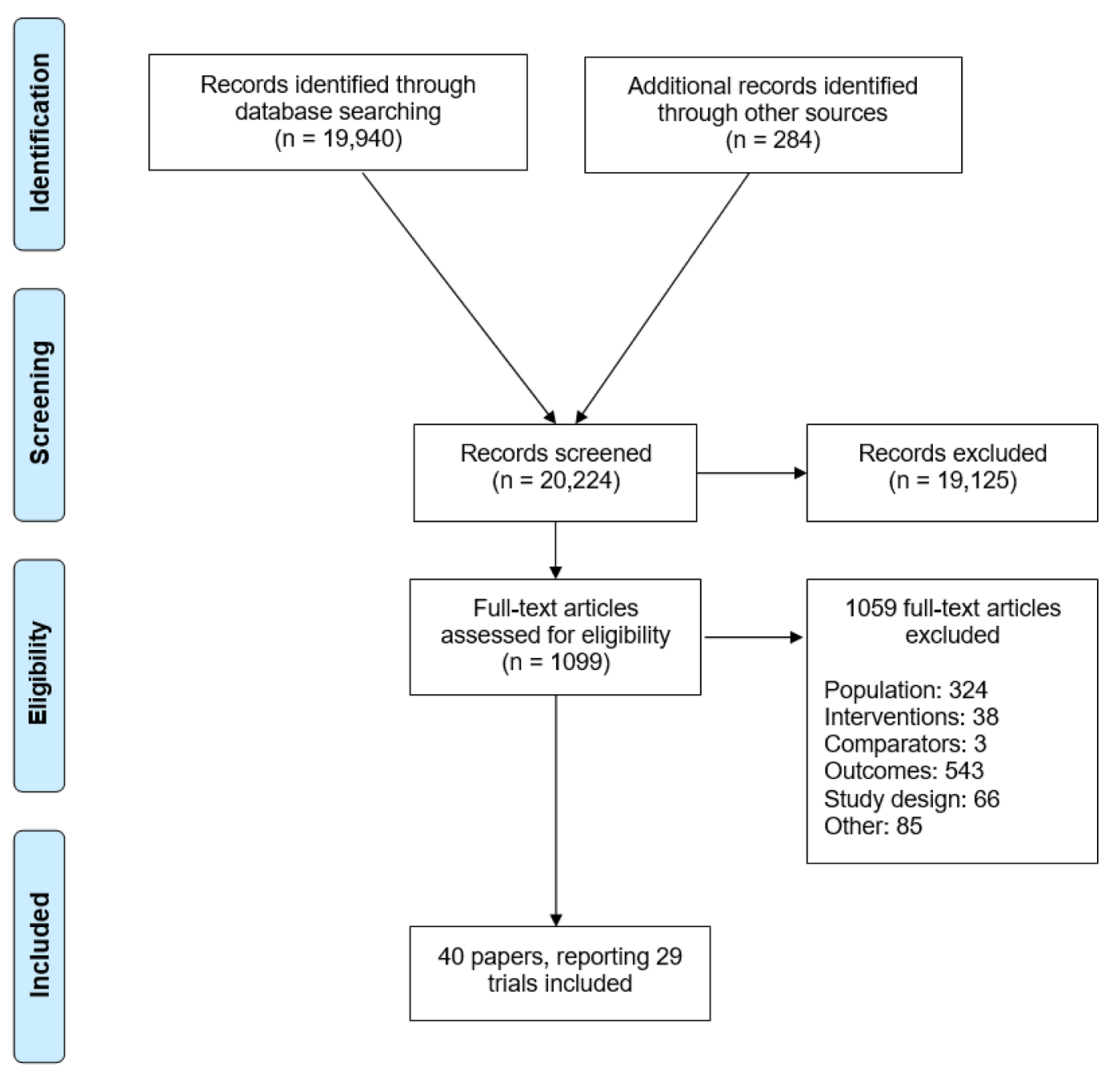
Study selection.

**Figure 2.**
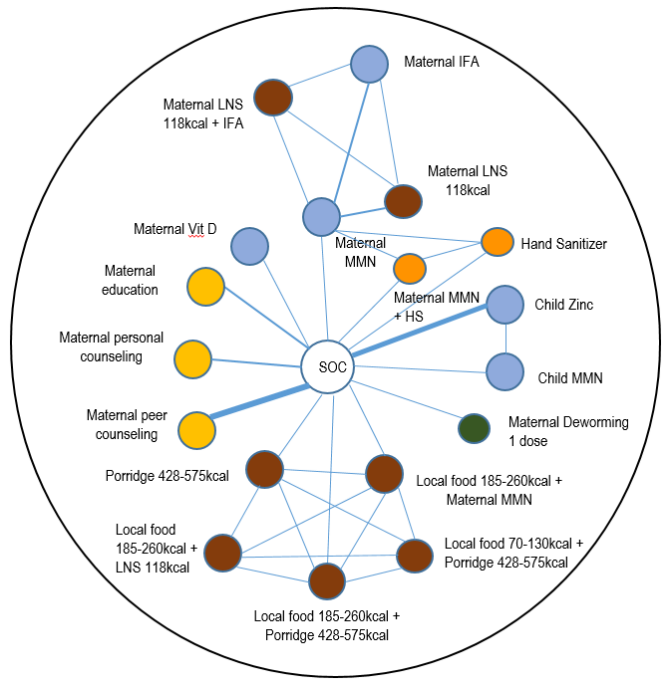
Overall network of the comparisons between interventions for exclusive breastfeeding period. Each node (circle) represents an intervention with each line representing a direct comparison between interventions (i.e. these interventions have been compared directly in a head-to-head randomized clinical trial). The width of the lines represents the numbers of trials with comparison in question. The white circle shows standard-of-care; blue circles represent micronutrient interventions; brown circles represent balanced energy protein or food supplements that are fortified and not; yellow circles represent education and counseling interventions; green circle represents deworming intervention; and orange circles represents WASH interventions. Fort, fortified; IFA, iron + folic acid; LNS, lipid based nutrient supplements; MMN, multiple micronutrients; SOC, standard of care; Vit, vitamin; HS, hand sanitizer.

We identified 20,224 abstracts from our database searches and hand searching of reference lists from published reviews ( Figure 1). Of these, 1099 studies underwent a full-text review, and 40 papers reporting on 29 trials met our inclusion criteria. In total, these trials pertained to 35,119 participants that were randomized to 73 unique interventions ( Figure 2). The list of the final subset is provided in Table 3, and the list of excluded studies ( *Extended data*, Supplementary Table 5) ^12^ is provided in the online appendix.

**Table 3.** The list of included studies.

Trial ID	Registry number	First author, year	Title
Acharya 2014 ^42^	NR	Acharya 2014	Randomized Control Trial of Kangaroo Mother Care in Low Birth Weight Babies at a Tertiary Level Hospital
Adu-Afarwuah 2016 ^35, 43^	NCT00970866	Adu-Afarwuah 2016	Small-quantity, lipid-based nutrient supplements provided to women during pregnancy and 6 mo postpartum and to their infants from 6 mo of age increase the mean attained length of 18-mo-old children in semi-urban Ghana: a randomized controlled trial
		Adu-Afarwuah 2017	Maternal supplementation with small-quantity lipid-based nutrient supplements compared with multiple micronutrients, but not with iron and folic acid, reduces the prevalence of low gestational weight gain in semi-urban ghana: A randomized controlled trial
Ashorn 2015A ^36, 44, 45^	NCT01239693	Ashorn 2015A	Supplementation of Maternal Diets during Pregnancy and for 6 Months Postpartum and Infant Diets Thereafter with Small-Quantity Lipid-Based Nutrient Supplements Does Not Promote Child Growth by 18 Months of Age in Rural Malawi: A Randomized Controlled Trial
		Ashorn 2015B	The impact of lipid-based nutrient supplement provision to pregnant women on newborn size in rural Malawi: a randomized controlled trial
		Adu-Afarwuah 2018	From the field: Improving fetal and infant growth in vulnerable populations
Boo 2007 ^46^	NR	Boo 2007	Short duration of skin-to-skin contact: effects on growth and breastfeeding
CARING trial ^47^	ISCRTN51505201	Nair 2017	Effect of participatory women's groups and counselling through home visits on children's linear growth in rural eastern india (caring trial): A cluster-randomised controlled trial
Feliciano 1994 ^48^	NR	Feliciano 1994	Seasonal and Geographical Variations in the Growth Rate of Infants in China Receiving Increasing Dosages of Vitamin D Supplements
Gathwala 2010 ^49^	NR	Gathwala 2010	Effect of Kangaroo Mother Care on physical growth, breastfeeding and its acceptability
Goodstart ^50, 51^	NR	Tomlinson 2011	An effectiveness study of an integrated, community-based package for maternal, newborn, child and HIV care in South Africa: study protocol for a randomized controlled trial
	NR	Tomlinson 2014	Goodstart: a cluster randomised effectiveness trial of an integrated, community-based package for maternal and newborn care, with prevention of mother-to-child transmission of HIV in a South African township
Habib 2015 ^52^	NCT01229579	Habib 2015	Zinc supplementation fails to increase the immunogenicity of oral poliovirus vaccine: A randomized controlled trial
Hamadani 2001 ^53^	NR	Hamadani 2001	Randomized controlled trial of the effect of zinc supplementation on the mental development of bangladeshi infants
JiVitA-3 ^54, 55^	NCT00860470	Christian 2016	Effects of prenatal multiple micronutrient supplementation on growth and cognition through 2 y of age in rural Bangladesh: the JiVitA-3 Trial
		West 2014	Effect of maternal multiple micronutrient vs iron-folic acid supplementation on infant mortality and adverse birth outcomes in rural Bangladesh: the JiVitA-3 randomized trial.
Kumbhojkar 2016 ^56^	NR	Kumbhojkar 2016	Kangaroo Mother Care (KMC): An Alternative to Conventional Method of Care for Low Birth Weight Babies
Le Roux 2013 ^57, 58^	NCT00996528	Le Roux 2013	Outcomes of home visits for pregnant mothers and their infants: a cluster randomized controlled trial
		Rotheram-Borus 2014	A Cluster Randomised Controlled Effectiveness Trial Evaluating Perinatal Home Visiting among South African Mothers/Infants
Locks 2016 ^59, 60^	NCT00421668	Locks 2016	Effect of zinc and multivitamin supplementation on the growth of Tanzanian children aged 6–84 wk: a randomized, placebo-controlled, double-blind trial
		Locks L 2015	Effect of zinc & multiple micronutrient supplements on growth in tanzanian children
Lonnerdal 2017 ^61^	NCT00970398	Lonnerdal 2017	Growth, Nutrition, and Cytokine Response of Breast- fed Infants and Infants Fed Formula With Added Bovine Osteopontin
LUCOMAI ^62^	NCT01977365	Nikiema 2017	Effectiveness of facility-based personalized maternal nutrition counseling in improving child growth and morbidity up to 18 months: A cluster-randomized controlled trial in rural Burkina Faso
MDIG ^63^	NCT01924013	Roth 2018	Vitamin D Supplementation in Pregnancy and Lactation and Infant Growth
Mofid 2017 ^39^	NCT01748929	Mofid 2017	A Double-Blind Randomized Controlled Trial of Maternal Postpartum Deworming to Improve Infant Weight Gain in the Peruvian Amazon
Osendarp 2002 ^64^	NR	Osendarp 2002	Effect of zinc supplementation between 1 and 6 mo of life on growth and morbidity of Bangladeshi infants in urban slums on the mental development of Bangladeshi infants
Ostadrahimi 2017 ^65^	NR	Ostadrahimi 2017	The effect of perinatal fish oil supplementation on neurodevelopment and growth of infants: a randomized controlled trial
PROCOMIDA ^38^	NCT01072279	Olney 2018	Procomida, a food-assisted maternal and child health and nutrition program, reduces child stunting in guatemala: A cluster-randomized controlled intervention trial
PROMISE EBF ^66– 68^	NCT00397150	Engebretsen 2014	Growth effects of exclusive breastfeeding promotion by peer counsellors in sub-Saharan Africa: the cluster-randomised PROMISE EBF trial
		Fadnes 2016	Effects of an exclusive breastfeeding intervention for six months on growth patterns of 4–5 year old children in Uganda: the cluster-randomised PROMISE EBF trial
		Tylleskar 2011	Exclusive breastfeeding promotion by peer counsellors in sub-Saharan Africa (PROMISE-EBF): a cluster-randomised trial.
RDNS ^37, 69^	NCT01715038	Dewey 2017	Lipid-based nutrient supplementation in the first 1000 d improves child growth in Bangladesh: a cluster-randomized effectiveness trial
		Mridha 2016	Lipid-based nutrient supplements for pregnant women reduce newborn stunting in a cluster-randomized controlled effectiveness trial in Bangladesh
Shafique 2016 ^40^	NCT01455636	Shafique 2016	Mineral- and vitamin-enhanced micronutrient powder reduces stunting in full-term low-birth-weight infants receiving nutrition, health, and hygiene education: A 2 × 2 factorial, cluster-randomized trial in bangladesh
Simondon 1996 ^41^	NR	Simondon 1996	Effect of early, short-term supplementation on weight and linear growth of 4-7-mo-old infants in developing countries: a four-country randomized triaI
Suman 2008 ^70^	NR	Suman 2008	Kangaroo mother care for low birth weight infants: a randomized controlled trial
Urban 2008 ^71^	NR	Urban 2008	Growth of infants born to HIV infected women when fed a biologically acidified starter formula with and without probiotics
Vazir 2013 ^72^	NR	Vazir 2013	Cluster-randomized trial on complementary and responsive feeding education to caregivers found improved dietary intake, growth, and development among rural Indian toddlers
Velaphi 2008 ^73^	NR	Velaphi 2008	Growth and metabolism of infants born to women infected with human immunodeficiency virus and fed acidified whey- adapted starter formulas

The trial characteristics of the included studies ( *Extended data*, Supplementary Table 6) ^12^ are provided in the online appendix. Of the 29 included trials, ten were cluster randomized trials (1156 clusters; 24,389 mother-infant dyads). The majority of trials were conducted in Southeastern Asian (n = 14) and African (n = 10) countries, and involved individual randomization (i.e. non-cluster trials, n = 19) and were open-label trials (n = 9). Several trials (n = 24) focused on a single domain of interventions, with micronutrient (n = 11) and food supplements (n = 9) being the most common intervention domains investigated. There were four trials that investigated interventions from two different intervention domains ^35– 39^, but the scope of these trials was still limited to nutritional (micronutrient and food) supplementations. There was one trial reporting on deworming study ^39^ and another on WASH intervention ^40^, and there were five trials on kangaroo care. There were 24 trials that investigated other intervention domains (non-kangaroo care trials), the median duration of interventions was 24 weeks (IQR: 12, 24 weeks). The kangaroo care trials entailed short follow-ups, with intervention durations that varied between one to two weeks.

The subject baseline characteristics are provided in the online appendix ( *Extended data*, Supplementary Table 7) ^12^. The median age of mothers at enrollment was 25.4 years (ranging from 21.8 to 29.8 years). For infants, the majority of trials enrolled participants from birth (after follow-up of the mother) or within the first month of life, except one trial ^41^ that investigated the effects of food supplements for an early weaning off breastfeeding enrolled subjects at 4 months of age (up to 7 months of age). The proportion of boys included in these trials was 51.3% on average, ranging from 36.6% ^40^ to 73% ^42^.

### Network meta-analysis on LAZ

**Figure 3.**
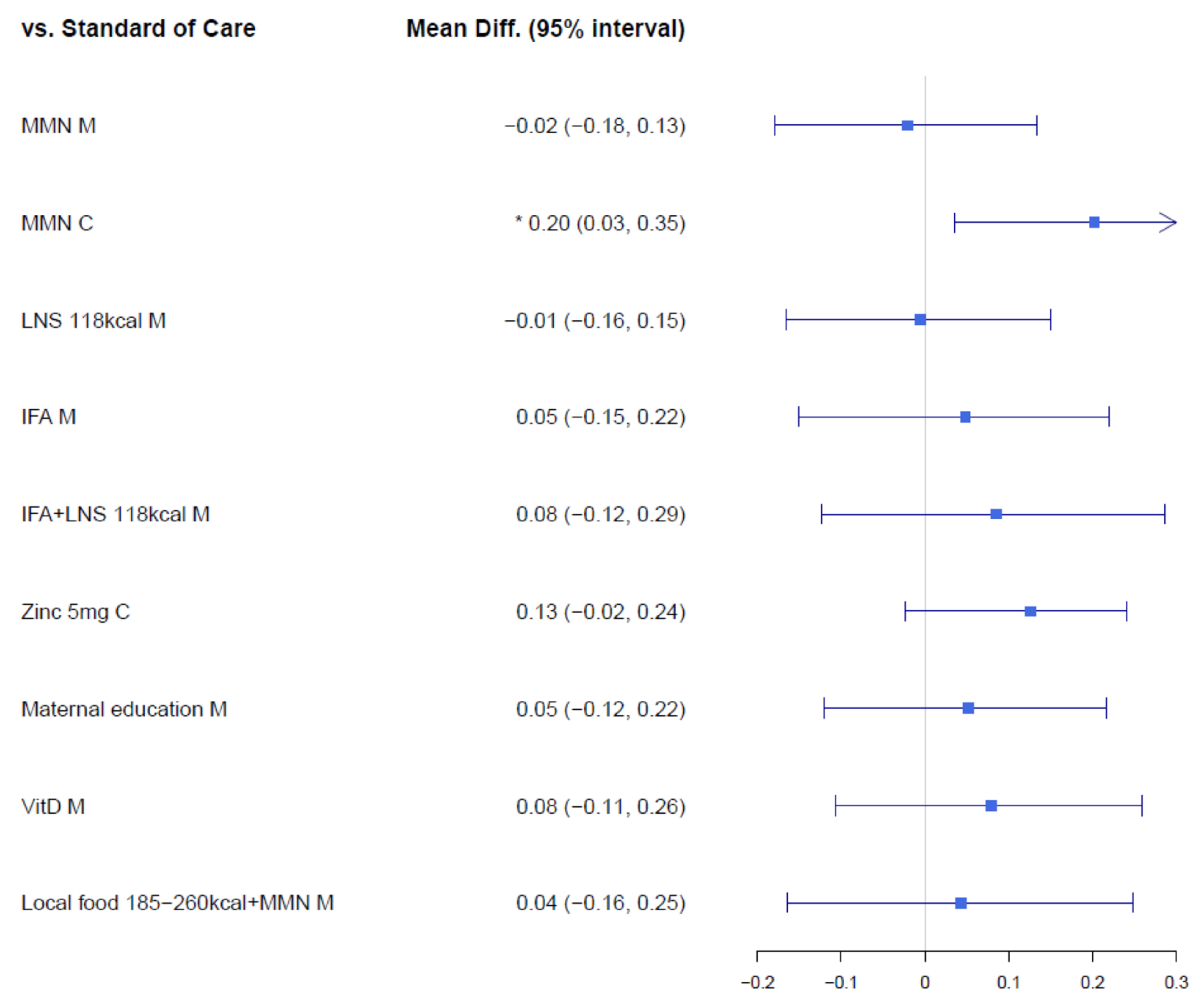
Forest plot for the effects of interventions on LAZ (mean difference in cm), cluster & non-cluster trials. Vit, vitamin; IFA, iron and folic Acid; LNS, lipid-based nutrient supplements; Fort, fortification; MMN, multiple micronutrients; M, maternal; C, child.

The LAZ network ( *Extended data*, Supplementary Figure 1) ^12^ included 18 trials consisting of 27,896 mother-infant dyads randomized to 52 intervention arms. The results of our primary analysis on LAZ that included both cluster and non-cluster randomized clinical trials are illustrated in Figure 3. Among micronutrient supplements, multiple micronutrients supplementation (MMN) provided to infants improved LAZ relative to standard-of-care (MMN-C, mean difference: 0.20, 95% CrI: 0.03, 0.35), whereas supplementing breasting mothers with MMN did not improve LAZ (MMN-M, mean difference: -0.02, 95% CrI: -0.18, 0.13). Compared to standard of care, other micronutrient supplements to infants, such as zinc 5 mg (zinc 5 mg C) showed a trend towards improved LAZ, but its CrIs overlapped the null of effect of 0.00 (Mean difference: 0.13; 95% CrI: -0.02, 0.24). Also, other micronutrients to breastfeeding mothers, such as iron and folic acid (IFA-M, mean difference: 0.05, 95% CrI: -0.15, 0.22) and vitamin D (Vit D-M: mean difference: 0.08, 95% CrI: -0.11, 0.26), did not improve LAZ in comparison to standard of care. Similarly, both food supplements and maternal education interventions did not improve LAZ; for instance, in comparison to standard-of-care, combination of IFA and 118 kcal of lipid-based nutrient supplements (IFA+LNS 118 kcal-M) showed a mean difference of 0.08 cm (95% CrI: -0.12, 0.29) for LAZ, where maternal education showed a mean difference of 0.05 cm (95% CrI: -0.12, 0.22 cm). No deworming or WASH interventions showed improvements on LAZ.

### Network meta-analysis on stunting

The stunting network ( *Extended data*, Supplementary Figure 3) ^12^ included 18 trials that consisted of 27,896 mother-infant dyads randomized to 52 intervention arms. The results of our primary analysis that included both cluster and non-cluster randomized clinical trials are illustrated in *Extended data*, Supplementary Figure 9 ^12^. While supplementation of zinc to infants (Zinc 5 mg-C, relative risk [RR]: 0.82, 95% CrI: 0.56, 1.18) showed a trend towards reduced risk of stunting, its CrI contained the null effect of 1.00. In fact, no interventions demonstrated any improvements towards reducing the risk of stunting.

### Sensitivity analyses on LAZ and stunting

Our sensitivity analyses were limited to individually (non-cluster) randomized clinical trials only. The network diagrams for LAZ ( *Extended data*, Supplementary Figure 2) ^12^ and stunting ( *Extended data*, Supplementary Figure 4) ^12^ can be found online along with forest plots ( *Extended data*, Supplementary Figures 10 and 11) ^12^ and cross-tables ( *Extended data*, Supplementary Table 9 and 10) ^12^. In our sensitivity analysis on LAZ, no interventions showed improvements for LAZ when compared to standard-of-care, and similarly for stunting, no interventions showed reduced risks for stunting. This is likely due to very few studies being available for sensitivity analyses; only nine trials were available for LAZ and stunting analyses.

### Kangaroo care

Five randomized clinical trials investigating the effects of kangaroo care on linear growth of newborns were included in the pairwise meta-analysis ^42, 46, 49, 56, 70^. The outcome reporting of these kangaroo care trials was limited to growth velocity of head circumference and length (cm per week). All kangaroo care trials were conducted in Southeastern Asian countries (i.e. India, Malaysia, and Nepal), in hospital settings and involved low birthweight neonates. Kangaroo care consisted of skin-to-skin contact between the mothers’ breasts, where infants in the control group were kept under either a warmer or incubator. The effects of kangaroo care on head circumference and length growth velocities are shown in *Extended data*, Supplementary Figures 12 and 13 ^12^, respectively. All studies except for Acharya 2014 ^42^ showed improvements in head circumference (Mean (SD): 31.5 (1.4) 95%CI -0.5, 0.6 ^46^; Kangaroo mother care (KMC): 0.75 cm vs conventional method of care (CMC): 0.49 cm p<0.001 ^56^;) and length (KMC: 0.99 cm vs CMC 0.70 cm p<0.001 ^56^). The pooled estimates of growth velocities for head circumference and length showed improvements for kangaroo care in comparison to the control. Relative to the control, kangaroo care showed an improved mean difference of 0.20 cm/week (95% CI: 0.09, 0.31 cm/week) for head circumference, and for length, a mean difference of 0.23 cm/week (95% CI: 0.10, 0.35 cm/week).

There is no blinding of participants in the kangaroo care studies, indicating a high performance bias in the studies. However, the impact of the expected degree of bias on the estimated treatment effect is difficult to assess ^74^.

### Qualitative summary of trials reporting on length and head circumference

There were twelve trials available for our qualitative summary ^38, 39, 41, 48, 52, 54, 57, 61, 64– 66, 72^. Of these trials, three were cluster randomized clinical trials that investigated interventions related to maternal education and breastfeeding promotion: Le Roux ^57^ Vazir ^72^, and PROMISE EBF ^66^ did not find differences in their maternal education and breastfeeding promotion interventions. In this three-arm trial conducted in India, mothers in the Complementary Feeding group (n = 202; 20 clusters) received nutrition education messages on breastfeeding and complementary feeding from community health workers (CHWs), and the mothers in the Complementary Feeding + Play group (n = 195; 20 clusters) received messages on psychosocial stimulation in addition to the same nutritional messages received by the women in the complementary feeding group (the control group received local standard of care; n = 202; 20 clusters). The mothers were approached by the trial investigators during pregnancy, and the interventions began when their child was three months old. By the age of six months, this trial found no differences in terms of length between the three groups (Mean ± SD: Control group: 64.2 ± 2.3; Complementary Feeding group: 64.4 ± 2.5 cm; and Complementary Feeding + Play group: 64.2 ± 2.3).

Additionally, nine trials investigated the effect of nutritional interventions (four trials on micronutrient supplements, three on food supplements, one on both, and one other for deworming) on the incidence of changes in head circumference, or changes in length ^38, 39, 41, 48, 54, 61, 64, 65, 72^. Of these nine trials, JiVitA-3 trial ^54, 55, 75^ and Ostadrahimi ^65^ provided supplements to mothers from pregnancy into postpartum, where the other five trials provided supplements to children. PROCOMIDA ^38^ provided food to the entire family. Ostadrahimi ^65^ enrolled pregnant women from the 20 ^th^ week of gestational age and were provided daily fish oil supplements (120 mg docosahexaenoic acid and 180 mg eicosapentaenoic acid) or placebo up to 1 month into the postpartum, with their child being followed-up up to six months of age. At the 6-month assessment of this trial, there were no differences found in neither length (mean difference: 0.12, 95% CI: -0.52, 0.76) or head circumference (mean difference: -0.03, 95% CI: -0.38, 0.30) between the fish oil and placebo groups. Mofid ^39^ found that deworming interventions provided to mothers who tested positive for soil-transmitted helminth infection at baseline had a positive impact on mean length gain (Mean difference: 0.8; 95% CI: 0.1, 1.4) and LAZ (mean difference:0.5; 95% CI: 0.2, 0.8) of infants at six months of age.

Three of the five trials investigated the effectiveness of micronutrient supplements administered directly to children ^48, 61, 64^. In Feliciano ^48^, three different dosages of Vitamin D supplements (daily dose of 100, 200, and 400 IU) were provided to Chinese infants from birth up to six months of age; at the 6-months assessment, differences in length between the three groups were observed. Another placebo-controlled trial ^64^ conducted in Bangladesh found that daily zinc supplements (5 mg) to children between the age of one month to six months did not change the length or head circumference.

There were two trials that explored the role of food supplements to children. Simondon *et al.* ^41^ was a multi-national trial (Congo, Sengal, Bolivia, and New Caledonia) that randomized four-month old infants to either cereal-based precooked porridge fortified with MMN or the control group consisting of local food. The mean consumption of supplement varied from 133 to 189 kcal/day. There were no differences in length (cm) between the supplemented and control groups in all four countries at six months of age. In Lonnerdal *et al.* ^61^, one-month old infants of non-breastfeeding mothers were randomized to receive regular formula or formula fortified with bovine osteopontin (65 or 130 mg/L). There were no differences in length or head circumference between children who were randomized to different formula groups. This trial also recruited infants whose mothers had expressed the desire to exclusively breastfeed up to six months of age and used this breastfeeding group as a non-randomized control. The breastfeeding group had a higher mean head circumference but similar length at six months of age.

## Discussion

Despite recent global achievements towards improved MNCH, the existing evidence on exclusive breastfeeding period interventions for linear growth remains unclear. Our study aimed to improve the current evidence base by assessing the comparative effectiveness of interventions across several domains: micronutrients, food supplements, maternal education, WASH, deworming, and kangaroo care. Both network meta-analysis and pairwise meta-analysis techniques were undertaken to appraise and synthesize findings from relevant studies reporting the desired outcomes for infants of age 0–6 months in LMICs (i.e. LAZ and proportion of stunted), and due to limited number of studies, length and head circumference were summarized qualitatively.

We found that MMN supplementation to infants (i.e. MMN-C) was the only intervention that showed important improvement for linear growth during the exclusive breastfeeding period. However, this finding was limited to only one trial in the study ^59^. Our analysis of kangaroo care also exhibited important improvements in growth in terms of increased head circumference and length growth velocity. However, kangaroo care interventions were excluded from the network meta-analysis and were analyzed separately via pairwise meta-analysis. This was due to the specific nature of this type of intervention, consisting of skin-to-skin contact between mothers’ breasts during a precise period for a limited duration (of between 1 and 6 weeks). In relation to this point was the observed heterogeneity in the intervention duration between included studies, generally, creating an added challenge when making comparisons across interventions. Deworming and WASH interventions did not show any improvements in both LAZ and stunting. 

 The main strength of this study was the use of network meta-analysis to assess the effectiveness of different interventions from a large network of evidence compared to standard-of-care ^76^. Previous reviews have focused only on intervention(s) within a single domain ( Table 1). We used a broad evidence base that included multiple interventions from different domains to simultaneously analyze all potential treatment options and make full use of the available evidence within a single analysis ^77, 78^. Additionally, appropriate statistical adjustments were made for clustering effects of cluster randomized clinical trials to enable the convergence of cluster and non-cluster trials for our network meta-analysis. Nevertheless, the narrow parameters of our PICOS criteria may have limited the breadth of our evidence base. Ethical and resource challenges associated with conducting clinical trials with neonates may have influenced investigators’ decision to undertake other non-randomized methodological approaches, such as observational studies. Additionally, since our population of interest focused on newborns living in LMICs, this prevented the inclusion of several trials conducted in non-LMICs. A number of studies assessed the effectiveness of long chain poly unsaturated fatty acids ^79– 81^; an intervention that has demonstrated some promise for improving linear growth in neonates compared to standard of care. As these trials were limited to high income settings, we were unable to incorporate this data into our analyses.

 In general, our analysis revealed that the existing evidence base for improving linear growth during the exclusive breastfeeding period is limited. Our scan and appraisal of the evidence resulted in a paucity of studies focused on this early life stage. The scarcity of evidence for this early life stage could be explained by several factors. Generally, clinical trials involving neonates are considerably more difficult to perform due to a range of ethical, physiological, pharmacometric, and economic challenges ^82^. Obtaining ethical clearance for enrolling neonates can be extremely tasking, particularly with the need to preserve equipoise between intervention arms through balancing risk factors across intervention groups ^82^. Such complexities can complicate both the study design and recruitment, especially as it pertains to trials conducted in resource scarce settings ^83^. These reasons may explain to why the current evidence base for exclusive breastfeeding period is limited.

More clinical trial research is needed for the EBF period. To enhance the quality of evidence, it will beneficial if trials in the future will utilize more efficient trial designs, such as adaptive trial designs, that can better manage the range of uncertainties that may be associated with investigations focused on neonates ^84, 85^. It is important for mothers and infants living in resource limited settings that our assessment of interventions is thorough and appropriate for diverse contexts and settings. This will be a critical step to achieve the global goal of achieving a 40% reduction in the number of stunted children <5 years by 2025 ^86^.

## Data availability

### Underlying data

All data underlying the results are available as part of the article and no additional source data are required.

### Extended data

Open Science Framework: Interventions to improve linear growth during exclusive breastfeeding life-stage for children aged 0–6 months living in low- and middle-income countries: a systematic review with network and pairwise meta-analyses. https://doi.org/10.17605/OSF.IO/T3JZQ ^12^

File ‘EBF period NMA - Supplementary tables and figures – v3.0’ contains the following extended data:

- Appendix 1. Literature search strategy. (Contains Supplementary Tables 1–3.)
- Appendix 2. Details to our statistical analysis.
- Appendix 3. List of included and excluded studies after full-text review. (Contains Supplementary Tables 4 and 5.)
- Appendix 4. Details of the evidence base. (Contains Supplementary Tables 6 and 7.)
- Appendix 5. Bias Assessment. (Contains Supplementary Table 8.)
- Appendix 6. The intervention networks for LAZ and stunting. (Contains Supplementary Figures 1–4.)
- Appendix 7. Primary analysis leverage and consistency plots. (Contains Supplementary Figures 5–8.)
- Appendix 8. Forest plots, cluster and non-cluster trials. (Contains Supplementary Figures 9–11.)
- Appendix 9. Forest plots for kangaroo care (Contains Supplementary Figures 12 and 13.)
- Appendix 10. Cross tables for LAZ and stunting. (Contains Supplementary Tables 9–12.)

### Reporting guidelines

Open Science Framework: PRISMA checklist for “Interventions to improve linear growth during exclusive breastfeeding life-stage for children aged 0–6 months living in low- and middle-income countries: a systematic review with network and pairwise meta-analyses.” https://doi.org/10.17605/OSF.IO/T3JZQ ^12^.

Data are available under the terms of the Creative Commons Attribution 4.0 International license (CC-BY 4.0).
