# Supplementary material for: Interventions to improve linear growth during exclusive breastfeeding life-stage for children aged 0-6 months living in low- and middle-income countries: a systematic review with network and pairwise meta-analyses
Source: Gates Open Res. 2020 Sep 24;3:1720. Originally published 2019 Dec 3. [Version 2] doi: 10.12688/gatesopenres.13082.2 (PMC7536351; doi:10.12688/gatesopenres.13082.2)
Supplement: Peer review history

# Peer review: Reviewer response for version 2

**DOI:** 10.21956/gatesopenres.14382.r29670

**Published:** 2020-10-05

**Authors:** James A. Berkley

**Affiliations:** 1 Centre for Geographic Medicine Research, Kenya Medical Research Institute (KEMRI)-Wellcome Trust Research Programme, Kilifi, Kenya; 2 University of Oxford, Oxford, UK

The changes made have improved readability and it is clearer that outcomes were assessed at 6 months of age. The issues I raised around timing of the interventions were understandably difficult to tackle because of heterogeneity, however this should be made clear as a limitation in the discussion since it is plausible that effects vary between conception and 6 months of age.

Are the rationale for, and objectives of, the Systematic Review clearly stated?

Yes

Is the statistical analysis and its interpretation appropriate?

Partly

Are sufficient details of the methods and analysis provided to allow replication by others?

Yes

Are the conclusions drawn adequately supported by the results presented in the review?

Partly

Reviewer Expertise:

Infection and nutrition in infants and children; clinical trials; cohort studies; anthropometry.

I confirm that I have read this submission and believe that I have an appropriate level of expertise to confirm that it is of an acceptable scientific standard.

# Peer review: Reviewer response for version 1

**DOI:** 10.21956/gatesopenres.14224.r28443

**Published:** 2020-02-19

**Authors:** Glenda Courtney-Martin

**Affiliations:** 1 Research Institute, The Hospital for Sick Children, Toronto, ON, Canada

The authors state that exclusive breastfeeding during the first six months of life is critical for linear growth. Although the evidence is strong to support the benefits of exclusive breastfeeding on linear growth, the evidence for other interventions is not as clear. Numerous published reviews have assessed the effectiveness of micronutrients, food supplements, deworming, maternal education and kangaroo care during the exclusive breastfeeding period but their scope has been limited to single intervention within a single domain. Because the determinants of linear growth for the exclusive breastfeeding period is multi-faceted there is a need to summarize the evidence from multiple domains. The study therefore uses a comprehensive literature review for multiple intervention domains of micronutrients, food supplements, deworming, maternal education, water sanitation and hygiene and kangaroo care with a goal to summarize their effects on linear growth during the first 6 months of life (exclusive breastfeeding period). The main outcomes of interest were LAZ, stunting, changes in linear growth and changes in head circumference. Network meta-analysis was used to analyze data except kangaroo care which was analyzed using pairwise meta-analysis. The main finding from this analysis was that micronutrient supplementation to infants was the only intervention that showed improvement in linear growth during the exclusive breastfeeding period.

 This is a valuable study and generally well designed. It can be improved in the following minor areas:

**Introduction:**

- The introduction could be tweaked to make it clearer. In the first paragraph it is not clear that linear growth is a clear benefit of exclusive breastfeeding during the first 6 months. For example “…is a critical life stage for early child development including linear growth.”

**Method:**

1. Clarify how the LAZ was calculated and if the same data was used to calculate LAZ among all trials.
2. There seems to be an error in reporting the length of the intervention among studies. For example on page 4/17 it reads “we excluded studies that did not report the effects of their respective interventions for at least three months”. However in table 3 under outcomes it states “At least one of the following outcomes (reported after at least 2 months, *except for Kangaroo care)”. Please clarify if this is 2 or 3 months.
3. At the bottom of page 4/17 the last sentence states “if any discrepancies occurred between the studies selected by the same reviewers, a third investigator provided arbitration”. Do you mean discrepancies in the review of the studies?
4. Top of second column page 4/17 - should this be subject characteristics rather than patient characteristics?
5. Table 3 should likely be table 2 and vice versa, since table 3 was written first in the text.
6. It is not clear in the methods when the respective outcomes were measured. Were they all measured before 6 months of life?
7. Also not clear why 2 or 3 months (depending on which one is accurate) was chosen as the minimum length of time for each intervention (except kangaroo care).  

**Results:**

1. Page 5/17 second column second paragraph the word “patients” is used rather than subjects?
2. What does CHW mean on page 9/17?

**Discussion:**

- Include a comment on whether the duration of the interventions were appropriate to see the desired outcomes.

**Title:**

- Substitute with for and in the title: “...a systematic review with network and pairwise meta-analysis...”.

Are the rationale for, and objectives of, the Systematic Review clearly stated?

Partly

Is the statistical analysis and its interpretation appropriate?

I cannot comment. A qualified statistician is required.

Are sufficient details of the methods and analysis provided to allow replication by others?

Yes

Are the conclusions drawn adequately supported by the results presented in the review?

Yes

Reviewer Expertise:

Nutrition.

I confirm that I have read this submission and believe that I have an appropriate level of expertise to confirm that it is of an acceptable scientific standard.

# Response

**Published:** 2020-08-20

**Authors:** Edward Mills

Dear Dr. Courtney-Martin:

Thank you for your thorough review of our manuscript. Our responses to your individual recommendations and comments are marked with bullets below.

Sincerely,

Edward J. Mills

The introduction could be tweaked to make it clearer. In the first paragraph it is not clear that linear growth is a clear benefit of exclusive breastfeeding during the first 6 months. For example “…is a critical life stage for early child development including linear growth.”

 

- *Thank you for your comment. We have revised the introduction* *to improve the readability. The goal of this paper was to see if there was any evidence of other interventions, such as micronutrients, food supplements, deworming, maternal education, and kangaroo care that impacted linear growth in children during the exclusive breastfeeding period. We have highlighted the benefits of exclusive breastfeeding in the article [A version of the article has been submitted to Gates Open Research].  *

Clarify how the LAZ was calculated and if the same data was used to calculate LAZ among all trials.

 

- *Thank you for the suggestion. We have updated the methods section to include the following:*

*“We used LAZ reported from the individual trials that were calculated using the WHO Child Growth Standards”.[Page 4, 4 ^th^ paragraph]*   

There seems to be an error in reporting the length of the intervention among studies. For example on page 4/17 it reads “we excluded studies that did not report the effects of their respective interventions for at least three months”. However in table 3 under outcomes it states “At least one of the following outcomes (reported after at least 2 months, *except for Kangaroo care)”. Please clarify if this is 2 or 3 months.

 

- *Supplementary Table 6 reports intervention duration of each trial. We have changed the text on page 4 to include “we excluded studies that did not report the effects of their respective interventions for at least **two** months” to keep it consistent with Table 2. *

At the bottom of page 4/17 the last sentence states “if any discrepancies occurred between the studies selected by the same reviewers, a third investigator provided arbitration”. Do you mean discrepancies in the review of the studies?

 

- *If any discrepancy occurred between the reviewers during abstract and full text screening as well as data extraction, a third investigator provided arbitration. * 

Top of second column page 4/17 - should this be subject characteristics rather than patient characteristics?

 

- *We have amended our article to include “subjects” in place of patients. * 

Table 3 should likely be table 2 and vice versa, since table 3 was written first in the text.

 

- *Thank you for the suggestion. We have amended the manuscript to include PICOS under Table 2 and list of included studies under Table 3. *

It is not clear in the methods when the respective outcomes were measured. Were they all measured before 6 months of life?

 

- *There is variation among the trials in terms of the time when the outcomes were measured.* *For the purpose of our analysis, we only included outcomes that were reported within 6 months of the child’s life. *

Also not clear why 2 or 3 months (depending on which one is accurate) was chosen as the minimum length of time for each intervention (except kangaroo care).

 

- *The goal was to capture desired outcomes that were reported within 6 months of the child’s age since the paper focused on the effect of exclusive breastfeeding for children aged 0-6 months. The highest length of time for intervention in the studies was 24 weeks.*

Page 5/17 second column second paragraph the word “patients” is used rather than subjects?

 

- *We have amended our article to include “subjects” in place of patients. *

What does CHW mean on page 9/17?

 

- *Thank you for pointing this out. It means community health workers. We have described the full abbreviation on the article. [Page 9 under qualitative summary]*

Include a comment on whether the duration of the interventions were appropriate to see the desired outcomes.

 

- *We have indicated under the discussion section of the article that there are observed heterogeneity in the intervention duration among included studies which created a challenge when making comparisons across interventions. *

Substitute with for and in the title: “...a systematic review with network and pairwise meta-analysis...”.

 

-  
*Thank you for the suggestion. We have changed the title of the article to “Interventions to improve linear growth during exclusive breastfeeding life-stage for children aged 0-6 months living in low- and middle-income countries: a systematic review with network and pairwise meta-analyses”.*

# Peer review: Reviewer response for version 1

**DOI:** 10.21956/gatesopenres.14224.r28319

**Published:** 2020-01-03

**Authors:** James A. Berkley

**Affiliations:** 1 Centre for Geographic Medicine Research, Kenya Medical Research Institute (KEMRI)-Wellcome Trust Research Programme, Kilifi, Kenya; 2 University of Oxford, Oxford, UK

- This is a very valuable analysis of the effects on stunting of interventions applied to mothers and infants in pregnancy and/or during the first six months of life. The methods used allow some comparisons between interventions to be made and aid interpretation of the limited data across the different type of interventions.
- Intervention in the target age group 0-6 months is of key importance given that it is a period of very rapid relative growth, exclusive breastfeeding is not practiced to 6 months of age in many low and middle income settings, and stunting seems to occur even when exclusive breastfeeding is reported.
- My main comments are around lack of analysis or reporting of duration of interventions and the age/timing of when they were applied.
- In the trials in more than one domain, infant supplementation (LNS etc.) was given after 6 months of age - hence these components do not always fall within the target age range, presumably some of these components were excluded and this could be specifically mentioned under trial characteristics.
- Timing - it would be helpful to readers to add the periods of pregnancy or during the first six months of life where interventions applied, and when LAZ was assessed as columns to Table 2.
- In figure 2, were the lower brown nodes (local food, porridge) administered to the infants or to the mothers? If mothers, then were they before, during or after pregnancy? For infants, from what age for zinc, MMN and foods? The timing (trimesters and say 0-2m/3-5m) and recipient (mother or infant) could be indicated by shape.
- 'Infant' rather than 'child' would be more appropriate throughout.
- Within the target period, there are differences in timing and age between the interventions and also potentially differences in plasticity in length or head growth with age. Kangaroo care applied in a selected LBW group at the lowest end of the infant age range when head and length growth is most rapid (and possibly most plastic) and was treated separately, but pre/post-partum and timing within the first 6 months could similarly be a determinant of response. If so, this would be very important for policy. Timing and duration of intervention could be examined as covariates in the network analysis vs SOC, and the estimated effects reported.
- For kangaroo care, the mean length and head circumference estimates are presented as absolute cm values rather than z scores and are presented as per week increment during a very short period of intervention. For the other interventions, the overall effect is reported despite varying duration. I appreciate the reasons for treating kangaroo care separately. However, it would be much more valuable to readers to report all results as changes per week over n weeks (or x weeks prepartum plus y weeks postpartum). For example, the foods given from age 4 months in some studies (a maximum of 2 months of intervention before assessment at 6 months), or formula milk at 1 month (for how long?) clearly differ in terms of both timing and duration from other interventions.
- Unless all studies other than kangaroo care reported measured LAZ at 6 months of age or another standardized time point, this would also need to be addressed in analysis, especially if any interventions went on for longer than the first 6 months of life.
- In the qualitative summary please specifically comment on blinding in the kangaroo care studies since these measurements are operator dependent and knowledge of the randomization at measurement could be a problem.
- Please replace '...showed a trend towards...' with '...was not statistically significant at the x percent alpha level...'. Zinc and MMN-m mentioned under Network meta-analysis on stunting do not look like borderline results. Overall, these would be more clear if presented as Forrest plots, similar to those under Network meta-analysis on LAZ.

Are the rationale for, and objectives of, the Systematic Review clearly stated?

Yes

Is the statistical analysis and its interpretation appropriate?

Partly

Are sufficient details of the methods and analysis provided to allow replication by others?

Yes

Are the conclusions drawn adequately supported by the results presented in the review?

Partly

Reviewer Expertise:

Infection and nutrition in infants and children; clinical trials; cohort studies; anthropometry.

I confirm that I have read this submission and believe that I have an appropriate level of expertise to confirm that it is of an acceptable scientific standard, however I have significant reservations, as outlined above.

# Response

**Published:** 2020-08-20

**Authors:** Edward Mills

Dear Dr. Berkley:

Thank you for your thorough review of our manuscript. Our responses to your recommendations and comments are marked with bullets below.

Sincerely,

Edward J. Mills

In the trials in more than one domain, infant supplementation (LNS etc.) was given after 6 months of age - hence these components do not always fall within the target age range, presumably some of these components were excluded and this could be specifically mentioned under trial characteristics.

 

- *Thank you for your comment. For the purpose of our analysis, we included outcomes that were reported within 6 months of age. Supplementary tables 6 and 7 report age of mother/child during enrolment and the intervention duration. *

Timing - it would be helpful to readers to add the periods of pregnancy or during the first six months of life where interventions applied, and when LAZ was assessed as columns to Table 2.

 

- *Supplementary Table 7 does report average gestational age at the time of recruitment. We have also updated the table to include average age of child (if applicable) at the time of enrolment. Supplementary Table 6 reports intervention duration of each trial. Information from both Table 6 and 7 can be used to gauge when LAZ was reported. The updated supplementary file can be found here: https://doi.org/10.17605/OSF.IO/T3JZQ. *

In figure 2, were the lower brown nodes (local food, porridge) administered to the infants or to the mothers? If mothers, then were they before, during or after pregnancy? For infants, from what age for zinc, MMN and foods? The timing (trimesters and say 0-2m/3-5m) and recipient (mother or infant) could be indicated by shape.

 

- *Interventions provided to mothers have been indicated as “maternal” on the nodes. Thus, the lower brown nodes show interventions that were administered to infants. Supplementary Table 7 indicates the ages of mother and child at enrolment as well as the average gestational age during recruitment. The network diagram was drawn from a treatment network generated from our Bayesian network meta-analysis tool which shows multiple treatment comparison among all the trials. Treatments have not been segregated according to duration for data analysis purposes. *

 'Infant' rather than 'child' would be more appropriate throughout.

 

- *Thank you for the suggestion. We decided to use the term “child” specifying the age range in order to be more specific. Infants usually indicate young children between the age of 0-1 year (https://www.cdc.gov/ncbddd/childdevelopment/positiveparenting/infants.html)*

 

Within the target period, there are differences in timing and age between the interventions and also potentially differences in plasticity in length or head growth with age. Kangaroo care applied in a selected LBW group at the lowest end of the infant age range when head and length growth is most rapid (and possibly most plastic) and was treated separately, but pre/post-partum and timing within the first 6 months could similarly be a determinant of response. If so, this would be very important for policy. Timing and duration of intervention could be examined as covariates in the network analysis vs SOC, and the estimated effects reported.

For kangaroo care, the mean length and head circumference estimates are presented as absolute cm values rather than z scores and are presented as per week increment during a very short period of intervention. For the other interventions, the overall effect is reported despite varying duration. I appreciate the reasons for treating kangaroo care separately. However, it would be much more valuable to readers to report all results as changes per week over n weeks (or x weeks prepartum plus y weeks postpartum). For example, the foods given from age 4 months in some studies (a maximum of 2 months of intervention before assessment at 6 months), or formula milk at 1 month (for how long?) clearly differ in terms of both timing and duration from other interventions.

 

- *Thank you for this comment. We acknowledge that there is substantial heterogeneity observed in the duration of the interventions and the timing of outcome assessments. To address the heterogeneity in trials, we employed random-effects model for our network meta-analysis. For kangaroo care studies, our outcomes reporting has been limited to growth velocity of head circumference and length (cm per week). Given the heterogeneity in timing and duration of interventions, we calculated pooled estimates of growth velocities. * 

Unless all studies other than kangaroo care reported measured LAZ at 6 months of age or another standardized time point, this would also need to be addressed in analysis, especially if any interventions went on for longer than the first 6 months of life.

 

- *The highest duration of intervention in the studies was 24 weeks (i.e. 6 months). Among those studies that reported an intervention duration of 24 weeks, some recruited mothers during pregnancy, while the children were recruited within a few days of birth. For the purpose of analysis, outcomes reported within the first 6 months of life were included. *

In the qualitative summary please specifically comment on blinding in the kangaroo care studies since these measurements are operator dependent and knowledge of the randomization at measurement could be a problem.

 

- *Thank you for pointing this out. We have added one paragraph under kangaroo care, which is as follows:*

 

*“There is no blinding of participants in the kangaroo care studies, indicating a high performance bias in the studies. However, the impact of the expected degree of bias on the estimated treatment effect is difficult to assess”. (https://www.bmj.com/content/368/bmj.l6802) [Page 9 under kangaroo care]* 

Please replace '...showed a trend towards...' with '...was not statistically significant at the x percent alpha level...'. Zinc and MMN-m mentioned under Network meta-analysis   on stunting do not look like borderline results. Overall, these would be more clear if presented as Forrest plots, similar to those under Network meta-analysis on LAZ.

 

- *We do not use terms such as “statistically significant or insignificant” to avoid dichotomization of scientific evidence. Zinc administered to infants was the only intervention that showed some promise on stunting, but its interval contained the null effect of 1. We have removed the effect of local food+MMN-m from the article. [Page 9, 1 ^st^ paragraph]. Forest plots on stunting have been included in the supplementary file (Figure 9 and 11). Available here: https://doi.org/10.17605/OSF.IO/T3JZQ. *
